# Efavirenz Interacts with Hormones Involved in Appetite and Satiety, Affecting Body Weight in Mice

**DOI:** 10.3390/ijms27020735

**Published:** 2026-01-11

**Authors:** Sandra Angélica Rojas-Osornio, Leticia Manuel-Apolinar, Minerva Crespo-Ramírez, Vladimir Paredes-Cervantes, Antonio Mata-Marín, José Molina-López, Miguel Pérez de la Mora, Dasiel Borroto-Escuela, Ricardo Martínez-Lara, Emiliano Tesoro-Cruz

**Affiliations:** 1 Departamento de Formación Básica Disciplinaria, Academia de Bioquímica Médica II, Escuela Superior de Medicina, Instituto Politécnico Nacional, Mexico City 11340, Mexico; srojaso@ipn.mx; 2 Unidad de Investigación Médica en Enfermedades Endocrinas, Hospital de Especialidades, Centro Médico Nacional “Siglo XXI”, Instituto Mexicano del Seguro Social, Mexico City 06720, Mexico; leticia.manuel@imss.gob.mx; 3 Division de Neurociencias, Instituto de Fisiología Celular, Universidad Nacional Autónoma de México, Mexico City 04510, Mexico; mcrespo@ifc.unam.mx (M.C.-R.); mperez@ifc.unam.mx (M.P.d.l.M.); 4 Laboratorio Central, Hospital de Especialidades “Dr. Antonio Fraga Mouret”, Centro Médico Nacional “La Raza”, Instituto Mexicano del Seguro Social, Mexico City 02990, Mexico; vlapace@hotmail.com; 5 Departamento de Infectología, Hospital de Infectología del Centro Médico Nacional “La Raza” IMSS, Mexico City 02990, Mexico; jamatamarin@gmail.com; 6 Unidad Periférica de Investigación Básica y Clínica en Enfermedades Infecciosas, Departamento de Salud Pública, División de Investigación, Facultad de Medicina, Universidad Nacional Autónoma de México, Mexico City 04510, Mexico; joseml@unam.mx; 7 Laboratorio de Patogenicidad Bacteriana, Unidad de Hemato-Oncología e Investigación, Hospital Infantil de México Federico Gómez, Facultad de Medicina, Universidad Nacional Autónoma de México, Mexico City 06720, Mexico; 8 Receptomics & Brain Disorders Lab, Instituto de Investigación Biomédica de Málaga y Plataforma en Nanomedicina–IBIMA Plataforma BIONAND, 29010 Malaga, Spain; dasiel@uma.es; 9 Department of Human Physiology and Physical Education and Sport Sciences, School of Medicine, University of Malaga, 29010 Malaga, Spain; 10 Department of Neuroscience, Karolinska Institutet, 17177 Stockholm, Sweden; 11 Unidad de Investigación Biomédica en Inmunología e Infectología, del Hospital de Infectología del Centro Médico Nacional “La Raza” IMSS, Mexico City 02990, Mexico; rml900_z@hotmail.com

**Keywords:** efavirenz, appetite, ghrelin, leptin, weight loss

## Abstract

Antiretroviral drugs are associated with increased body weight and metabolic disorders. Fat gain and insulin resistance are commonly associated with abdominal obesity in people with HIV (PWH). There is currently an open ongoing discussion about how antiretroviral therapy affects body weight and its significance in hunger–satiety circuit alteration. Until now, the impact of the drug on this circuit has not been explored. This study aimed to assess the hormones involved in appetite and satiety regulation in the serum and hypothalamus after efavirenz (EFV) administration in mice. EFV (10 mg/kg) and distilled water (1.5 μL/kg) (control group) were orally administered for 36 days to CD1 mice. Body weight and food intake were determined throughout treatment. At the end of the treatment, the metabolic profile (glucose, triglycerides, cholesterol) was assessed, and leptin, soluble receptor of leptin (sOB-R), and ghrelin were measured in serum; moreover, we evaluated the expression of growth hormone secretagogue receptor 1a (GHS-R1a), neuropeptide Y receptor 1 (NPYR1), and leptin in the hypothalamus, and a sucrose preference test (SPT) was conducted. Outcomes showed an increase in serum ghrelin and the expression of GHS-R1a and NPYR1 receptors in the hypothalamus, coinciding with an increase in appetite and preference for sucrose in mice in the EFV group. Furthermore, serum leptin, sOB-R, and the free leptin index (FLI) showed that hunger is not related to a lack of satiety. Despite increased food intake, a reduction in body weight was observed, and triglyceride and cholesterol levels were increased. According to our findings, mice treated with EFV showed a decrease in body weight, despite increased food intake resulting from appetite stimulation, which is caused by specific compounds, hormones, and neural signals acting on the brain’s hunger centres, primarily in the hypothalamus, promoting eating behaviours. However, further studies are necessary to investigate the mechanisms of EFV’s effects on energy expenditure.

## 1. Introduction

HIV reduces appetite and causes infections that force the body to burn extra calories in an attempt to fight them. Before effective treatments became available and when old antiretroviral treatments (ARTs) were used, people with HIV (PWH) suffered from wasting syndrome, losing more than 10% of their body weight and becoming very weak because of the redistribution of body fat secondary to mitochondrial alterations, which clinically manifests as lipoatrophy [1].

Currently, combination antiretroviral therapy (cART) prevents weight loss and helps PWH live longer and healthier lives. Although classic lipodystrophy mainly occurs with older HIV drugs, newer HIV treatments are associated with alterations in body weight [2]. Previous research has revealed heterogeneity between ART regimens in terms of weight gain following ART initiation among previously ART-naïve PWH. On the other hand, greater weight gain was observed among PWH taking newer ART-based regimens (dolutegravir, bictegravir, tenofovir alafenamide, tenofovir disoproxil fumarate, emtricitabine, and efavirenz), although the extent varied among drugs. In addition, greater weight gain is noted among women than among men [3].

In this regard, several studies on the effects of ART on body weight have revealed that an increase in body weight is associated with alterations in biochemical profiles, mainly hyperglycaemia and hypertriglyceridemia, and enhances metabolic disorders such as insulin resistance, which is commonly associated with abdominal obesity in PWH [4,5,6,7].

Recent studies have suggested that contemporary ART regimens contribute to weight gain; however, the underlying mechanisms are unclear [8].

In previous research, we reported that chronic administration of efavirenz (EFV) dysregulated *Tph2* in the brain’s serotonergic areas, altering body weight and food intake in healthy CD1 mice [9]. Nevertheless, the mechanism underlying the possible alteration of the hunger–satiety circuit has not been studied.

EFV is a nonnucleoside reverse transcriptase inhibitor (NNRTI) and is most commonly used because of its efficacy and acceptable pharmacokinetics. However, the secondary effects associated with its use are numerous, including alterations in the central nervous system (CNS), such as impaired concentration, a reduction in quality sleep, insomnia, nightmares, dizziness, nervousness, anxiety, depression, psychotic behaviour, suicidal ideation, and seizures [10,11]. Some studies have reported that EFV is widespread throughout the entire rat brain, including the cerebral cortex, corpus callosum, basal forebrain, globus pallidus, hippocampal formation, striatum, caudoputamen, corticospinal tracts, thalamus, and hypothalamus [12]. Dysfunction in energy metabolism in the brain, especially in the cerebral cortex, striatum, and hippocampus, after the administration of EFV was observed in both clinical studies and murine models [13,14,15].

During the feeding process, the brain detects alterations in energy stores and triggers metabolic and behavioural responses designed to maintain energy balance. Energy homeostasis is controlled mainly by neuronal circuits in the hypothalamus and brainstem, whereas the reward and motivation aspects of eating behaviour are controlled by neurons in the limbic regions and cerebral cortex. Leptin, insulin, and metabolic hormones related to fat stores control body weight through long-term effects on feeding and energy expenditure [16,17].

Given the potential role of EFV in hunger and satiety, this study aims to investigate the hypothalamic hormones involved in these signals after chronic oral EFV administration in CD1 mice and its relationship with body weight, food intake, and metabolic profiles. However, since our model uses healthy mice to isolate the pharmacological effect of EFV from HIV infection variables, we recognise that the absence of viral load and chronic inflammation limits the direct translation of these findings to human patients.

## 2. Results

### 2.1. Body Weight and Metabolic Profile

**Figure 1:**
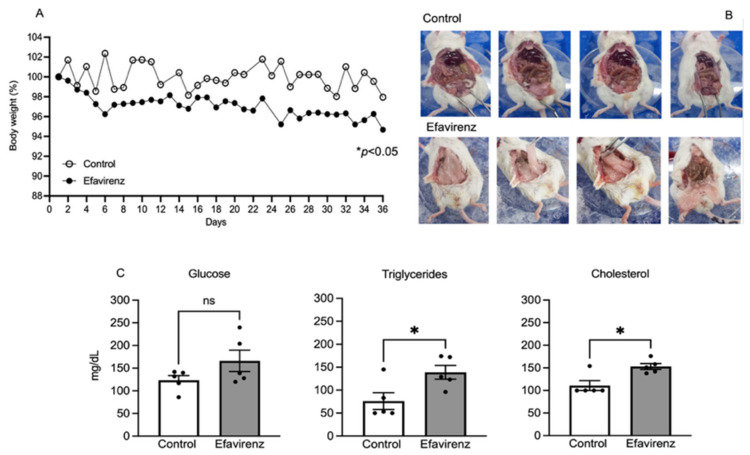
EFV effects on body weight in mice. (**A**) shows a significant decrease in body weight in the EFV group (F_35,540_ = 1.470; * *p* < 0.05; *n* = 28). (**B**) shows increased abdominal fat accumulation in mice compared with the control group; however, mice treated with EFV appeared thinner (*n* = 4, randomised). Additionally, (**C**) shows an increase in triglyceride (Welch’s *t* test, F_4,4_ = 1.481; * *p* < 0.05) and cholesterol (Welch’s *t* test, F_4,4_ = 2.581; * *p* < 0.05) levels in the EFV group. However, glucose concentration did not significantly increase (F_4,4_ = 5.250; *p* > 0.05) (*n* = 5 mice) (ns = not statistically significant).

During 36 days of EFV administration, body weight was monitored daily. We observed a significant decrease in bodyweight in mice in the EFV group from the beginning to the end of the administration period (one-way ANOVA, post hoc Tukey test, F_35,540_ = 1.470; *p* < 0.05) compared to the control (one-way ANOVA, post hoc Tukey test, F_35,630_ = 1.098; *p* > 0.05) (Figure 1A). At the end of the treatment, mice in the EFV group showed an accumulation of visceral fat, despite having a lower weight (Figure 1B). Regarding metabolic profiles, we detected elevated triglyceride (F_4,4_ = 1.481; *p* < 0.05) and cholesterol (F_4,4_ = 2.581; *p* < 0.05) levels in mice in the EFV group; however, glucose concentration did not significantly increase (F_4,4_ = 5.250; *p* > 0.05) (Figure 1C).

### 2.2. Orexins and Eating Behaviour

**Figure 2:**
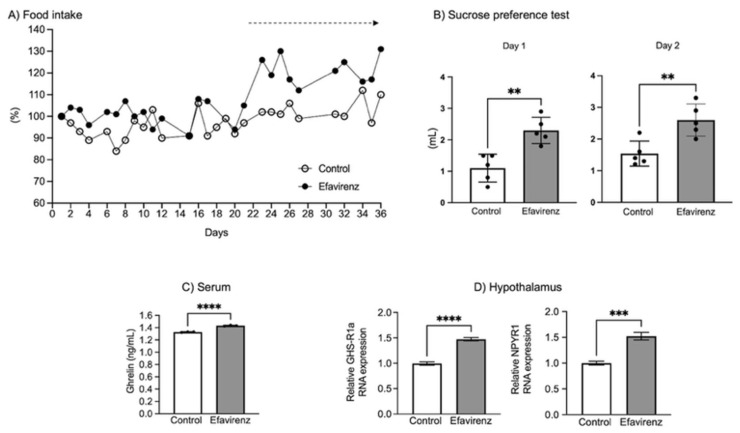
EFV effects on appetite. Food intake throughout 36 days of treatment did not show significant differences between the groups (EFV: F_24,35_ = 0.6709; *p* > 0.05; control: F_24,30_ = 0.5191; *p* > 0.05, *n* = 28); however, we observed that greater food consumption occurred in the group of mice treated with EFV from day 20 onwards (**A**) (black arrow). At the end of the treatment, two groups of five mice were submitted to the sucrose preference test (SPT) and mice treated with EFV showed a greater preference for sucrose vs. compared to control mice (day 1: Welch’s *t* test F_4,4_ = 1.114; ** *p* < 0.01; day 2: Welch’s *t* test F_4,4_ = 1.646; ** *p* < 0.01) (*n* = 5 mice) (**B**). Regarding orexigenic hormones, serum ghrelin (**C**) showed a significant increase (ghrelin: F_2,2_ = 1.333; **** *p* < 0.00001, *n* = 3) in mice in the EFV group. (**D**) shows an increase in GHR-1a (F_3,3_ = 6.705; **** *p* < 0.0001, *n* = 3) and NPYR1 (F_4,3_ = 1.628; *** *p* < 0.001, *n* = 3) expression in the hypothalamus in the EFV group versus the control group.

During the 36 days of the experiment, food intake was monitored daily. Although food intake was not significantly different (EFV: F_24,35_ = 0.6709; *p* > 0.05; control: F_24,30_ = 0.5191; *p* > 0.05), we observed that greater food consumption occurred in the group of mice treated with EFV from day 20 onwards (Figure 2A). At the end of the treatment, five animals from each group were subjected to the sucrose preference test (SPT). Mice in the EFV group showed an increased preference for sucrose compared with control mice (day 1: Welch’s *t* test F_4,4_ = 1.114; *p* < 0.01; day 2: Welch’s *t* test F_4,4_ = 1.646; *p* < 0.01) (*n* = 5 mice). Although the sucrose preference test is typically used to assess anhedonia, with the expectation that animals will prefer the sucrose solution, mice in the EFV group showed an even greater preference (Figure 2B). Moreover, at the end of the treatment and after 6 h of fasting, hormones related to hunger were measured in both groups. Serum ghrelin levels significantly increased (F_2,2_ = 1.333; *p* < 0.0001) (Figure 2C), and in the hypothalamus, GHS-R1a (F_3,3_ = 6.705; *p* < 0.0001) and NPYR1 (F_4,3_ = 1.628; *p* < 0.001) receptor expression increased in the EFV group (Figure 2D).

### 2.3. Leptin and Satiety

**Figure 3:**
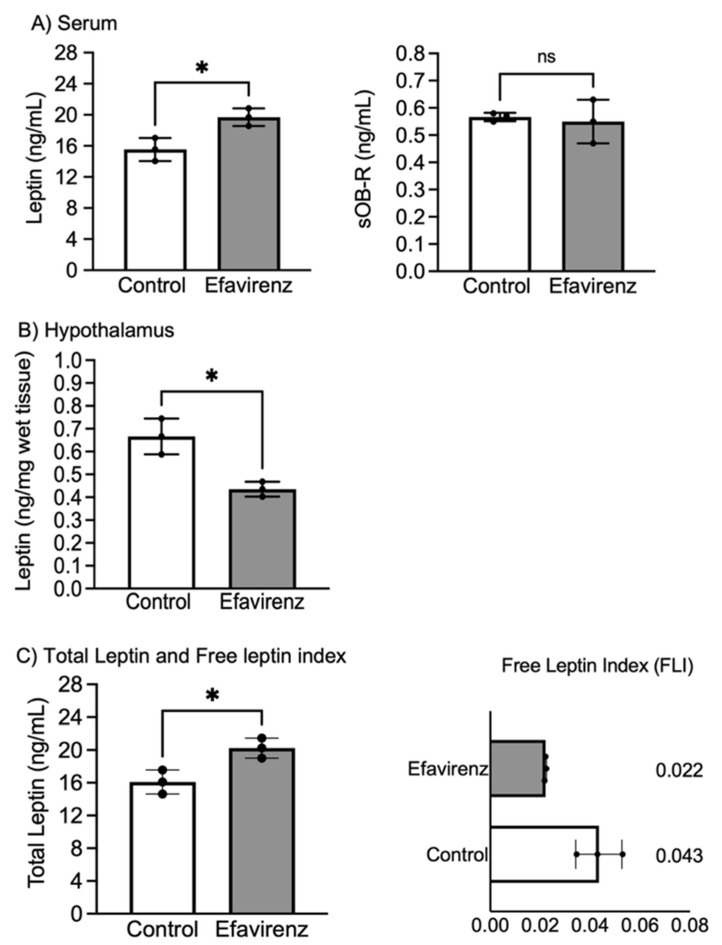
EFV effects on hormones related to satiety. (**A**) shows an increase in serum leptin in the EFV group vs. the control group (Welch’s *t* test F_2,2_ = 1.708; * *p* < 0.05, *n* = 3). However, no changes were detected in sOB-R in mice in the EFV group vs. the control group (Welch’s *t* test F_2,2_ = 27.43; *p* = 0.07, *n* = 3, ns: *p* > 0.05) (Figure 3A). (**B**) shows a decrease in leptin levels in the hypothalamus of mice in the EFV group compared to the control group (F_2,2_ = 5.754; * *p* < 0.05, *n* = 3). (**C**) shows total leptin and the free leptin index (FLI); the FLI was determined by summing circulating leptin and leptin bound to its receptor. Mice in the EFV group showed an increase in total leptin (F_2,2_ = 1.460; * *p* < 0.05, *n* = 3). The FLI was calculated as the ratio of hypothalamic leptin to total leptin (**C**). These data indicate that leptin signalling is adequate, since an increase in receptor-bound leptin could lead to a lack of satiety.

At the end of the treatment and after 6 h of fasting, hormone-related satiety was measured in both the EFV and control groups. Serum leptin levels significantly increased (F_2,2_ = 1.708; *p* < 0.05); however, soluble leptin receptor (sOB-R) levels did not differ between the EFV and control mice (F_2,2_ = 27.43; *p* = 0.07) (Figure 3A), while in the hypothalamus, leptin levels decreased in mice in the EFV group (F_2,2_ = 5.754; *p* < 0.05) (Figure 3B). Furthermore, we observed that total leptin (serum leptin plus leptin bound to the sOB-R) in EFV mice was higher than that in the control mice, but interestingly, the free leptin index was not greater in the EFV-treated group (Figure 3C).

## 3. Discussion

This study provides evidence on the relationship between metabolic dysfunction and antiretroviral drugs. In the case of EFV, metabolic dysfunction can affect hypothalamic function, potentially influencing hunger and satiety. Moreover, these mechanisms of weight gain or loss are controversial in PWH. On the other hand, no direct clinical studies have specifically evaluated hunger or satiety among people taking EFV. Hence, some individuals receiving EFV treatment may experience increased appetite and weight gain, whereas others might experience decreased appetite and weight loss [18]. In this regard, in the case of PWH, the presence of the virus alters their defence system. HIV replication and the ensuing inflammatory response cause a catabolic form of weight loss. The persistently high turnover of T cells and the removal of billions of virions daily, with associated ongoing inflammation, significantly elevate the basal metabolic rate, resulting in increased energy expenditure [19]. Persistent inflammation influences appetite, and one of the early reported benefits of ART initiation is increased hunger, presumably due to rapid control of viremia and inflammation [20]. Particularly in those who are starting treatment, the drug improves their health status by causing weight gain, but at the expense of exacerbating other factors affecting the infected individual [19,20].

In addition, it has also been reported that some people who experience neurotoxic effects possess the CYPB26 polymorphism. In individuals with a CYP2B6 variant that triggers reduced enzyme activity (e.g., the CYP2B6*6 allele), the drug is metabolised more slowly, leading to higher-than-normal blood concentrations and potentially an increased risk of adverse drug reactions or neurotoxic effects. The variability in drug blood levels caused by this polymorphism helps explain why some people experience side effects (like neurotoxicity or weight gain) while others on the same dosage do not [21]. In this regard, the presence of certain CYP2B6 polymorphisms results in less effective drug clearance, elevated blood levels, and consequently a higher likelihood of experiencing dose-dependent side effects. Excess weight gain on newer ARTs has emerged as a major clinical concern, leading to increasingly safe and potent ART agents being developed for the millions of PWH globally [21].

In animal models without viral infection, EFV primarily causes neuropsychiatric issues, neuroinflammation, developmental delays, and metabolic changes, often linked to its effects on the central nervous system (CNS) and cholesterol metabolism, impairments in short-term memory and spatial learning, anxiety and depression-like behaviours, dysregulation of the *Tph2* gene, and alterations in serotonin (5-HT) levels in certain brain structures such as the amygdala [9,12,15,22]. Moreover, EFV exhibits a long elimination half-life (~40–55 h), with plasma concentrations reaching a steady state within ~6–10 days of daily dosing. Sustained exposure beyond this period allows for both pharmacokinetic stabilisation and the development of downstream physiological responses, including enzymatic induction and metabolic signalling alterations. The 36-day treatment duration in this study therefore encompasses both the attainment and maintenance of steady-state exposure, providing sufficient time for early metabolic/hormonal adaptations. The suitability of the chosen duration is supported by translational data indicating measurable regulatory effects within this timeframe. This period thus aligns with the study’s objective of evaluating changes in body weight, appetite-regulating hormones, and metabolic parameters [23,24].

In the present study, the potential role of EFV in regulating hunger and satiety was studied in healthy CD1 male mice due to previous findings related to a decrease in body weight despite high food intake. To find a possible explanation for this controversial result, we assessed the levels of the hormones involved in appetite and satiety regulation, both in serum and in the hypothalamus after 36 days of chronic oral EFV administration. Moreover, it is important to mention that our model uses healthy mice to isolate the pharmacological effect of EFV from HIV infection variables.

The regulation of food intake is mediated by a complex mechanism in the hypothalamus, which is responsible for regulating hunger and satiety signals. These signals allow energy homeostasis to be achieved through specific structures, such as the lateral nucleus (responsible for the sensation of hunger) and the ventromedial nucleus (responsible for managing and controlling the sensation of satiety during food intake) [25].

Ghrelin is an orexigenic hormone that regulates feeding, the sleep–wake cycle, metabolism, and glucose homeostasis in humans and rodents. Ghrelin functions by binding to its receptor, the growth hormone secretagogue receptor 1a (GHS-R1a), which is widely expressed inside and outside the brain [26]. In the brain, this hormone enhances the activity of the lateral hypothalamic nucleus. However, it has been suggested that deacyl ghrelin is involved in the regulation of energy homeostasis because of its ability to cross the blood–brain barrier (BBB) and induce increased neuronal activity in the hypothalamic arcuate nucleus [27].

In our study, mice treated with EFV did not increase their food intake until day 20, the point at which their intake differed to that of the control mice, which corresponded with the increase in serum ghrelin levels at the end of the treatment, as well as increased GHS-R1a and NPYR1 expression in the hypothalamus (Figure 2A,C,D). During the sucrose preference test (SPT), we observed that mice in the EFV group showed an increased liking for sucrose (Figure 2B), which could be related to the high 5-HT levels seen in the amygdala after chronic EFV treatment in CD1 mice, as recently reported in [9]. Connections between the amygdala and hypothalamus enable 5-HT to influence the decision to initiate or terminate eating. In the amygdala, 5-HT modulates the emotional aspects of eating, connecting affective factors with the homeostatic mechanisms of the hypothalamus. Consequently, it plays a role in eating disorders, obesity, and food anxiety [28]. In turn, the amygdala is involved in modulating the emotional and motivational aspects of eating. In this sense, we believe that 5-HT is significant in the present study as a key neuromodulator in the regulation of hunger and satiety. In addition, the amygdala is also part of this circuit because it integrates emotional signals with the control of food intake. In the amygdala, 5-HT regulates the emotional balance of food (pleasure, aversion, and anxiety associated with eating) through 5-HT1A and 5-HT2C receptors [29].

Leptin’s role is to manage and control the sensation of satiety during food intake. Leptin levels indicate that nutritional stores are adequate. These levels decrease under conditions in which the nutritional status is suboptimal and increase under conditions in which energy stores are in excess, such as in obesity. Leptin crosses the BBB through saturated transport, circulating through circumventricular organs, e.g., the arcuate hypothalamic nucleus, subfornical organ, and area postrema [30].

According to our results, we observed that serum leptin levels increased in mice treated with EFV, but the levels decreased in the hypothalamus. However, when leptin is released into the blood from fat cells, it can circulate freely or bind to its receptor. The free fraction travels to the brain, where it crosses the BBB to act on receptors within the central nervous system, affecting appetite. Although we detected low leptin levels in the hypothalamus in EFV-treated mice (Figure 3B), this reduction could be due to negative feedback caused by the high ghrelin levels (Figure 2C). Furthermore, the FLI indicates that leptin signalling is adequate, since an increase in receptor-bound leptin can lead to a lack of satiety. The sOB-R binds to free leptin in the blood. This binding complex regulates appetite and metabolism, modulating levels of circulating leptin and affecting its action. The sOB-R reduces the amount of leptin available to bind to its cellular receptors; if free leptin decreases, the sOB-R can influence leptin signalling, meaning that higher sOB-R levels are associated with lower leptin signalling. The ratio of free leptin to total leptin can be an indicator of leptin resistance, which could lead to feelings of hunger even when the body has sufficient fat [31].

According to our results, no increase in the sOB-R was observed in the EFV-treated group, and although serum leptin was higher than in the control group, the hypothalamic response did not increase. Additionally, the FLI was low (0.022), which suggests that circulating leptin is active on its receptor and is not a factor that affects the sensation of hunger.

The relationship between leptin levels and lipoatrophy in PWH is controversial. Some studies have reported lower leptin levels in patients with lipoatrophy [32,33]. In contrast, others have reported that leptin in plasma is unlikely to play a major role in the genesis of lipoatrophy [34]. Studies have shown that both high and low food intake can influence leptin secretion, independent of the effect of adiposity [35].

Based on this information and according to our preliminary qualitative findings, we observed that serum leptin increased and body weight decreased, although abdominal fat accumulation was observed in mice treated with EFV (Figure 1A,B and Figure 3A).

Weight loss observed in healthy mice treated with EFV (Figure 1A) could be related to a decrease in their muscle or bone mass. Bone mineral density (BMD) loss and fat gain is common in PWH, particularly after initiating combination antiretroviral therapy (cART). Recently, Zhang et al. (2023) and Liang et al. (2024) reported not only greater BMD loss but also muscle loss in Chinese men treated with lamivudine (3TC)–tenofovir disoproxil fumarate (TDF)–efavirenz (EFV) [36,37].

In addition, this treatment could also lead to lipolysis, triggering abdominal fat accumulation (Figure 1B) due to an increase in triglycerides and cholesterol (Figure 1C) and a high glucose level in an attempt to compensate for the energy expenditure resulting from metabolic wear and tear in adipose and muscle tissue caused by EFV.

Moreover, Barroso et al. (2024) reported metabolic effects of first-line efavirenz, emtricitabine, and tenofovir disoproxil fumarate (EFV/FTC/TDF) in a single-tablet regimen in asymptomatic antiretroviral-naïve PWH treated for at least one year [38]. They observed an increase in plasmatic levels of glucose, total cholesterol, and triglycerides, demonstrating that this regimen causes subclinical metabolic alterations [38].

Although we did not determine fat, muscle, and bone mass percentages in mice using bioimpedance, recent reports have shown that this drug interferes with adipogenesis and muscle and bone loss in humans [36,37]. However, it would be interesting to measure these parameters to identify the most likely reason for weight loss and to compare the results with females, since differences in body weight by gender and age have been reported.

Limitations: This study has several limitations. Deacyl ghrelin was not measured; no comparison was made with females; hormones were not measured throughout the treatment period, inhibiting assessment of their behaviour over time; the percentage of fat, muscle, and bone mass was not determined by impedance; and in adipose tissue, we did not identify whether there was adipocyte hypertrophy. In addition, we acknowledge the loss of statistical power as a limitation due to the small number of mice in each test.

## 4. Materials and Methods

### 4.1. Mice and Ethical Aspects

Healthy, 12-to-14-week-old adult CD1 male mice (*n* = 56), 28 per group, weighing 40–44 g, were maintained in the Animal Facility of the Centro Médico Nacional “Siglo XXI”, Instituto Mexicano del Seguro Social (IMSS). At this age, mice are considered mature adults, fully developed physically and sexually; their physical growth is complete, and they exhibit adult social and reproductive behaviours. In addition, they have reached their final body size and weight, and their musculature is fully developed [39].

The mice were fed a commercial diet (5008, Lab-Diet. Hayward, CA, USA) (standard diet) and drinking water ad libitum. The mice were handled in accordance with the Mexican Official Standard (NOM-062-ZOO-1999, revised in 2015) [40] for the care and use of laboratory animals. The National Scientific Research Committee (IMSS) approved all of the experimental procedures, and a euthanasia method designed to cause minimal pain and distress under controlled conditions (12/12 h dark–light cycle; lights on 8:00–20:00 h; temperature of 21 ± 1 °C) was used (licence number R-2021-785-057). This study was conducted in accordance with the guidelines in the Guide for the Care and Use of Laboratory Animals established by the Mexican Animal Welfare and Ethical Authorities [40].

### 4.2. Pharmacological Treatment

The mice were randomly divided into two groups of twenty-eight animals each. One group received distilled water (1.5 μL/kg) (control group), and the other group received EFV (SUSTIVA^®^ tablets, 600 mg, by Bristol-Myers Squibb Pharma, Princeton, Nueva Jersey, EE. UU (10 mg/kg for 36 days) [9,15]. Distilled water and EFV were orally administered to the mice using an oral gavage needle (3.0 mm diameter, 1.2 mm curve and 55 mm length for the 18 G syringe, Ketu Store, made in China), attached to a syringe. Drug volumes were adjusted to the animal weights to prevent the toxic effects of the drug, and the animals were weighed daily. EFV was administered orally once a day for 36 days (chronic administration) at a dosage of 10 mg/kg, as reported by Romão et al. (2011) [15], on the basis of the doses used for human therapy (dose: 600 mg daily) as reported by Apostolova et al. (2015) [41], and as reported in previous studies with murines by Rojas-Osornio et al. (2024) [9] and Streck et al. (2008) [42].

### 4.3. Sucrose Preference Test

At the end of the treatment, five animals from each group, selected via systematic randomisation, were subjected to the sucrose preference test (SPT). The SPT was performed following the method described by Liu et al. (2018) [43].

A 2% (wt/vol) fresh sucrose solution was used. The solution was prepared fresh before the experiment and allowed to warm to room temperature (22 °C ± 1 °C) 1 h before being introduced to the animals each day and kept at 4 °C for no longer than 24 h.

The SPT is considered to selectively reflect the animal’s capacity to experience hedonic pleasure evoked by the sucrose solution (liking). Accordingly, a reduced sucrose preference in response to stress or other neural perturbations is typically interpreted as anhedonia in its most stringent definition of “loss of pleasure”.

### 4.4. Animal Handing and Collection of Biological Samples

Food intake was monitored daily and measured using a digital scale (Lab-Tech, Guadalajara, Mexico). The amount of food consumed per cage was weighed daily at 2:00 p.m. On the same day that EFV was initiated, 400 g of food was provided, and the amount of food given stayed the same for the entire 36-day period. At the end of EFV treatment, both study groups were subjected to euthanasia. Twenty-three animals from each group were anaesthetised (pentobarbital, 25 mg/kg, i.p.) to obtain whole blood through cardiac puncture. The blood was centrifuged at 3500 rpm/15 min/4 °C, and each serum sample obtained was stored at −20 °C until use. Later, nine animals per group were quickly decapitated, their brains were excised promptly from the skull, and the region of interest (hypothalamus) was rapidly dissected, frozen on dry ice, and stored at −80 °C until further analyses.

### 4.5. Serum Measurements

Leptin, sOB-R, and ghrelin levels of sera from nine mice, selected via systematic randomisation, were measured using an enzyme-linked immunosorbent assay (ELISA) kit (MyBioSource^®^, San Diego, CA, USA), and the measurements were performed in triplicate according to the manufacturer’s protocol. The plate was read spectrophotometrically using an ELISA reader (Thermo ScientificTM, Waltham, MA, USA) at 450 nm.

The mouse leptin solid-phase sandwich ELISA is designed to measure the amount of the target bound between a matched antibody pair. Briefly, a target-specific antibody was precoated in the wells of the supplied microplate. Samples, standards, and controls were then added to these wells and bound to the immobilised (captured) antibody. The sandwich was formed by adding the second (detector) antibody, and a substrate solution (TMB) that reacts with the enzyme–antibody–target complex to produce a measurable signal at 450 nm was added.

A mouse sOB-R ELISA kit was used to perform the double-sandwich ELISA. The precoated antibody was a mouse sOB-R monoclonal antibody, and the detection antibody was a polyclonal antibody labelled with biotin. The samples and biotin-labelled antibody were added to the wells of the ELISA plate, which was subsequently washed with PBS. Afterwards, avidin–peroxidase conjugates were added to the ELISA wells in order, and the TMB substrate was used for colour detection after the reactants were thoroughly removed through washing with PBS. TMB turns blue in peroxidase catalysis and yellow under the action of acid. The colour intensity and the testing factors of the samples were positively correlated.

The mouse GHRL (ghrelin) ELISA has high sensitivity and excellent specificity for the detection of ghrelin. It is based on the competitive ELISA detection method. The microtiter plate provided in this kit was precoated with the target. During the reaction, the target in the sample or standard competes with a fixed amount of target on the solid-phase support for sites on the biotinylated detection antibody specific to the target. Excess conjugated and unbound samples or standards were removed from the plate, and HRP–streptavidin (SABC) was added to each microplate well and incubated. The TMB substrate solution was subsequently added to each well. The enzyme–substrate reaction was terminated by adding a sulfuric acid solution, and the colour change was measured spectrophotometrically at 450 nm [44].

Serum leptin, sOB-R, and ghrelin concentrations are expressed in ng/mL.

### 4.6. Hypothalamus Measurements

A mouse leptin solid-phase sandwich ELISA was used (MyBioSource, San Diego, CA, USA). Briefly, the brain tissue of three mice was homogenised in PBS (pH 7.5) using a sonicator (Thermo Fisher Scientific, Waltham, MA, USA) (2 cycles of 30 s each) and centrifuged for 15 min at 5000 rpm and 4 °C. Leptin quantification was performed in triplicate according to the manufacturer’s protocol, and the plate was read spectrophotometrically with an ELISA reader (Thermo Scientific TM, Waltham, MA, USA) at 450 nm. Leptin in the wet tissue (hypothalamus) is expressed in ng/mg.

### 4.7. RT–PCR Analysis of GHSR-1a and NPYR1 mRNA Expression in the Hypothalamus

GHSR-1a and NPYR1 expression was evaluated using RT–PCR. At the end of EFV treatment, one pool of six hypothalamus samples was analysed after RNA extraction with 1 mL of TRIzol. RNA was separated with 0.2 mL of chloroform by centrifugation at 12,000× *g* for 10 min. The aqueous phase was recovered, and RNA samples were precipitated with 0.5 mL of isopropyl alcohol. The RNA pellet was recovered by centrifugation at 12,000× *g* for 15 min and then washed with 70% ethanol–DEPC water, air-dried at room temperature, and redissolved in water. The final RNA concentration was estimated by spectrophotometry (Nanodrop 2000, Thermo Fisher, Waltham, MA, USA). First-strand synthesis was performed in a total volume of 20 μL with 2 μg of RNA, random primers, dNTP mix, and adequate buffer conditions for the multiScribe Reverse Transcriptase (High-Capacity cDNA Reverse Transcription Kit, Applied Biosystems, Waltham, MA, USA).

Pairs of oligonucleotides yielding a Tm of 60 °C were used to amplify the GHSR-1a, NPYR1, and β-actin genes with products of 449, 73, and 374 bp, respectively. The primers used were as follows: GHSR-1a F5′GCGCTCTTCGTGGTGGGCATCT 3′, GHSR-1a R5′GTGGCGCGGCATTCGTTGGT3′; NPYR1 F5′TCTTCTCTGCCCTTCGTGATC3′, NPYR1 R 5′TGAACGCCGCAAGTGATACA3′. RT–PCR was performed in a 20 μL reaction mixture containing 1 μL of cDNA, 10 pM each of the forward and reverse primers, and GoTaq 2X (Promega, Madison, WI, USA).

The conditions for GHSR-1a, NPYR1, and β-actin gene amplification through PCR were as follows: 95 °C denaturation for 5 min; 35 cycles of denaturation at 95 °C for 1 min; alignment at 60 °C for 1 min; extension at 72 °C for 1 min; and a final extension at 72 °C for 7 min. A 374 bp product of the β-actin gene was used as an endogenous control for sample normalisation with the oligonucleotides β-actin F5′CCTGGTATGGAATCCTG-TG3′ and β-actin R5′TTGTAAAGAAAGGGTGTAAA3′. All the PCR products were visualised by electrophoresis on 2% agarose gel stained with ethidium bromide, and densitometry was performed using the Gel Doc EZ System (Bio-Rad, Laboratories, Inc., Hercules, CA, USA) [43].

### 4.8. Metabolic Marker Analysis

Glucose levels were determined using FreeStyle Optium Xceed glucometer test strips (Abbot Diabetes Care Ltd., OYL, Witney, Oxfordshire, UK), and cholesterol and triglyceride concentrations in 40 µL of blood were determined using reagent strips with the aid of CardioChek equipment (PTS diagnostics CardioChek plus 2700 professional analyser, Indianapolis, IN, USA). The sera from five mice were selected via systematic randomisation.

### 4.9. Statistical Analysis

The statistical analyses were performed using GraphPad Prism 6.0 statistical software (La Jolla, CA, USA). The distribution of the values of the studied parameters was tested for normality using the Shapiro–Wilk test.

Body weight was analysed using two-factor repeated-measures ANOVA, followed by the Tukey post hoc test. Metabolic profile data (glucose, triglycerides, cholesterol) were analysed using unpaired *t* tests with Welch’s correction to determine differences between the experimental group and the control group. Food intake was analysed by unpaired *t* tests with Welch’s correction. SPT, serum ghrelin, GHSR-1a, and NPYR1 in the hypothalamus were analysed using unpaired *t* tests with Welch’s correction to determine differences between the experimental and control group. Serum leptin, the sOB-R, and hypothalamus leptin were analysed using unpaired *t* tests with Welch’s correction to determine differences between the experimental and control group. All data were expressed as the mean ± standard error (S.E.M.) of three independent triplicate assays. Statistical significance was set at *p* < 0.05.

## 5. Conclusions

The data presented here suggest that chronic EFV consumption in healthy mice leads to increased hunger as a result of elevated serum ghrelin levels and increased expression of GHS-R1a and NPYR1 receptors in the hypothalamus. Satiety does not seem to be affected; despite experiencing weight loss, the increase in food intake could be due to appetite stimulation caused by specific compounds, hormones, or neural signals, acting on the brain’s hunger centres, primarily in the hypothalamus, and promoting eating behaviours.

However, further studies are necessary to investigate the mechanisms underlying EFV’s effects on energy expenditure.

## Data Availability

The original contributions presented in this study are included in the article. Further inquiries can be directed to the corresponding author.
